# Ultrasound-guided synovial tissue biopsy for people with rheumatoid arthritis: a micro-costing study

**DOI:** 10.1093/rap/rkaf011

**Published:** 2025-01-29

**Authors:** Sainan Chang, Felice Rivellese, Katherine Payne, Zoë Ide, Anne Barton, Costantino Pitzalis, Sean P Gavan

**Affiliations:** Manchester Centre for Health Economics, Division of Population Health, Health Services Research and Primary Care, School of Health Sciences, Faculty of Biology, Medicine and Health, University of Manchester, Manchester, UK; Department of Rheumatology, Mile End Hospital, Barts Health NHS Trust and NIHR Barts Biomedical Research Centre, London, UK; Centre for Experimental Medicine and Rheumatology, Queen Mary University of London, London, UK; Manchester Centre for Health Economics, Division of Population Health, Health Services Research and Primary Care, School of Health Sciences, Faculty of Biology, Medicine and Health, University of Manchester, Manchester, UK; NIHR Manchester Biomedical Research Centre, Manchester University NHS Foundation Trust, Manchester Academic Health Science Centre, Manchester, UK; Patient Researcher, University of Manchester, Manchester, UK; NIHR Manchester Biomedical Research Centre, Manchester University NHS Foundation Trust, Manchester Academic Health Science Centre, Manchester, UK; Versus Arthritis Centre for Genetics and Genomics, Centre for Musculoskeletal Research, Division of Musculoskeletal and Dermatological Sciences, School of Biological Sciences, Faculty of Biology, Medicine and Health, University of Manchester, Manchester Academic Health Science Centre, Manchester, UK; Department of Rheumatology, Mile End Hospital, Barts Health NHS Trust and NIHR Barts Biomedical Research Centre, London, UK; Centre for Experimental Medicine and Rheumatology, Queen Mary University of London, London, UK; Manchester Centre for Health Economics, Division of Population Health, Health Services Research and Primary Care, School of Health Sciences, Faculty of Biology, Medicine and Health, University of Manchester, Manchester, UK

**Keywords:** cost, health economics, precision medicine, resource, rheumatoid arthritis, synovial biopsy

## Abstract

**Objective:**

Identify and quantify the resource use and cost per patient of performing a US-guided synovial tissue biopsy for people with RA within a routine healthcare setting.

**Method:**

A micro-costing study was performed from a healthcare system perspective (National Health Service England). A service pathway conceptual model described how the procedure will be embedded within routine care to inform management decisions. Consumables were estimated from existing standard operating procedures for synovial biopsies. Staff time was estimated by expert input (clinical rheumatologist and patient). The time for tissue sample acquisition was obtained from data within the biopsy-driven R4RA trial. Unit costs were sourced from historic purchase prices, published salary scales and public-facing list prices. One-way sensitivity analysis identified key drivers of total cost per patient. Scenario analyses explored the cost impact if less senior healthcare staff performed the biopsy or if an additional outpatient appointment was required to identify joint suitability.

**Results:**

The total cost of US-guided synovial tissue biopsy was £356.24/patient (best-case estimate: £185.30; worst-case estimate: £812.46). The key driver of total cost was the time taken to perform tissue sample acquisition. The total cost was lower if a registrar performed the biopsy (£294.24) and higher if an additional outpatient appointment was required for joint selection (£438.98).

**Conclusion:**

Interventions requiring synovial tissue samples to inform treatment selection or prognosis are emerging. The findings can inform cost parameters for future cost-effectiveness analyses. These results will help resource allocation and clinical decisions to improve the value of treatments for RA.

Key messages

- Strategies to improve rheumatoid arthritis with synovial tissue biopsies are emerging for routine care.
- Healthcare providers now need evidence of the resources required and cost to deliver these biopsies.
- Understanding these resource requirements will support effective biopsy-based procedures to benefit people with rheumatoid arthritis.

## Introduction

There is growing international interest in identifying and revealing sources of heterogeneity affecting outcomes and treatment response for people with RA [1, 2]. Biopsy and subsequent analysis of synovial tissue samples presents one promising mechanism to reveal this heterogeneity between patients [3]. Recommendations by the EULAR have highlighted the potential scope for synovial biopsy-informed management in both research and care settings [4]. However, synovial tissue biopsies are not performed for RA within the current standard of care, which limits the scope for this intervention strategy to improve routine management decisions. A reliable estimate of the likely resources required and associated costs incurred to perform this procedure in a healthcare system will be valuable for decision makers and rheumatology care providers to help translate synovial tissue biopsy from the research environment into routine care settings.

Management of RA involves active use of conventional synthetic DMARDs, biologics and targeted agents to prevent long-term irreversible damage [5]. The inflammatory disease process leads to swelling in the synovial tissue surrounding patients’ joints [6]. Emerging clinical research has started to identify biomarkers within the synovial tissue that can be helpful in informing management decisions for RA [7, 8]. For example, biomarkers may be prognostic by helping to identify those people most likely to experience long-term severe active disease due to multidrug resistance [9, 10]. In such cases, these individuals may benefit from rapid escalation of treatment to mitigate the harm from exposure to earlier lines of ineffective therapies. Alternatively, biomarkers may be predictive and help inform the likely magnitude of response to alternative treatments available for RA [9]. For example, the absence of B cell signatures within synovial tissue samples may correspond with improved response to treatments that do not deplete B cells [11, 12].

Protocols to perform synovial tissue biopsies are now well-established, reflecting international consensus [13]. US is used to guide joint selection and tissue extraction. Most importantly, the biopsy procedure is safe and tolerated by people with RA [14, 15]. However, consideration must now be given to the necessary resources to embed this procedure within current pathways of care. These resources comprise the consumables to perform the biopsy, along with the time incurred by the healthcare workforce to undertake the biopsy and discuss the procedure or results within a shared decision-making setting.

Micro-costing is a useful method to establish the cost of new healthcare intervention strategies before they enter routine care [16]. This method takes a bottom-up approach to understand how new interventions will be delivered within current pathways of care, quantify the magnitude of resource items required and assign unit costs to those resource items [17]. Micro-costing has been used effectively by previous studies to estimate the cost of new testing strategies for people with RA [18]. A micro-costing analysis of the synovial tissue biopsy will be helpful to address the specific gap in evidence by making explicit the necessary resources required to perform this procedure within a routine care setting and the likely upfront cost incurred to rheumatology care providers. Therefore, this study aimed to identify and quantify the resource use and cost per patient of performing a US-guided synovial tissue biopsy for people with RA within a routine healthcare setting.

## Methods

This study reports a bottom-up micro-costing analysis of a US-guided synovial tissue biopsy for people with RA. The unit of analysis was an individual patient. A healthcare system perspective [National Health Service (NHS) England] was used (price year: 2022–23). The time horizon was defined as the period from discussing the biopsy with a patient during a routine outpatient visit until the eventual treatment decision (≈1 week). Costs were not discounted because the time horizon was <12 months [19].

### Defining the biopsy pathway

**Figure 1.**
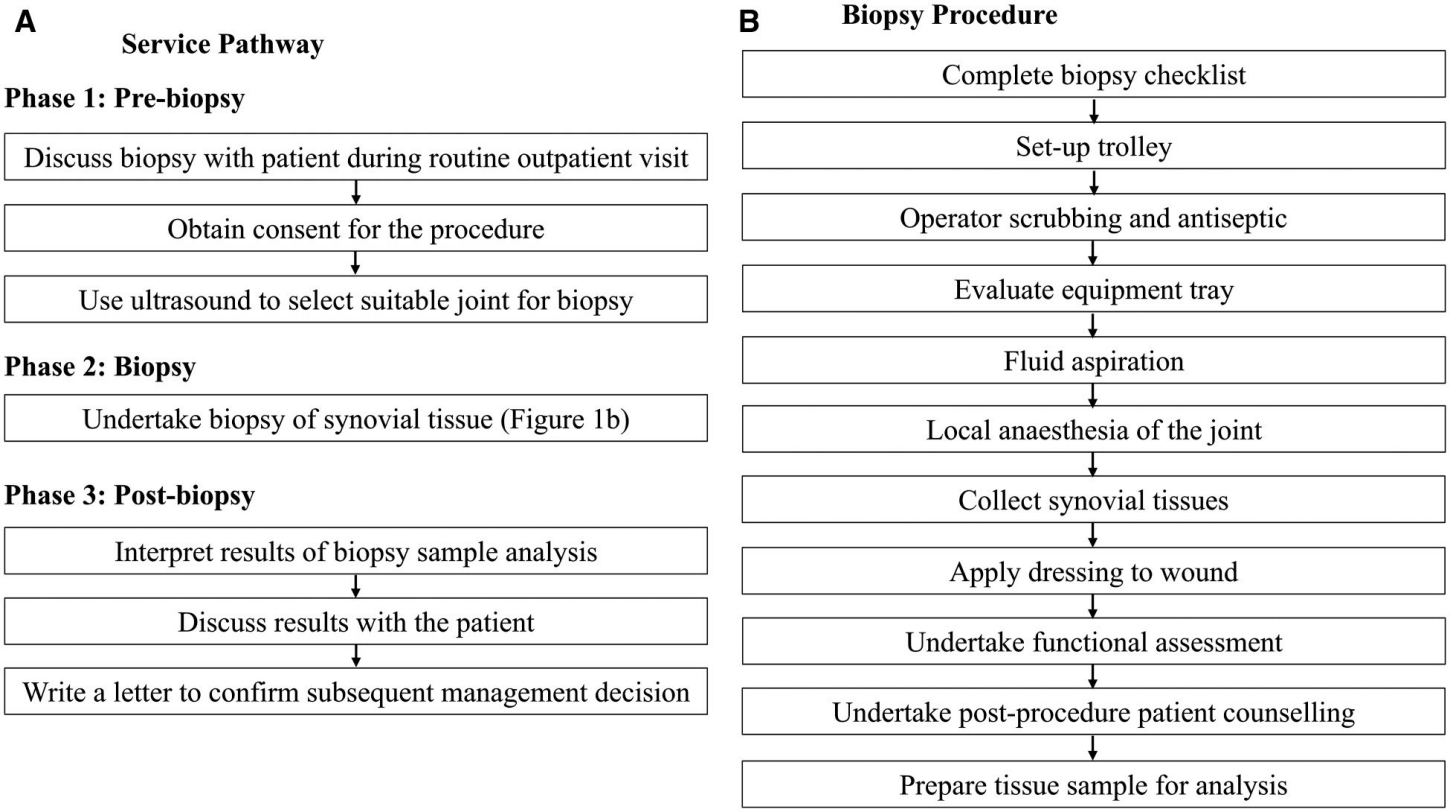
Conceptual model of the (**A**) US-guided biopsy service pathway and (**B**) biopsy procedure

A service pathway conceptual model was developed to define how the synovial tissue biopsy procedure will be embedded within routine care (see Fig. 1) [20]. This service pathway illustrated the sequence of events that take place between the initial routine outpatient appointment and an eventual prescribing decision. The pathway was divided into three phases: pre-biopsy, biopsy and post-biopsy treatment decision. The standard operating procedure (SOP) from the R4RA trial was used to define the necessary steps for the biopsy procedure [12]. Expert input from clinicians experienced with managing people with RA (F.R., C.P. and A.B.) and a patient collaborator (Z.I.) with experience living with RA were used to define the steps required to discuss the suitability of the biopsy within a routine outpatient setting and to inform a final prescribing decision. Expert input was obtained during two online videocalls with all individuals. During these videocalls, a diagram of the service pathway was developed iteratively until a group consensus was reached to ensure face validity.

During the pre-biopsy phase (see Fig. 1A), which happens during a routine outpatient visit, time is required to inform patients about the potential benefits and risks of the procedure, provide information to obtain informed consent and establish the presence of synovitis using a US scan to determine which joint is most suitable for biopsy. For the biopsy procedure, which is generally undertaken during a subsequent outpatient visit, Fig. 1B illustrates the sequence of events that occur between completing a biopsy checklist to preparing the tissue sample for analysis. During the post-biopsy treatment decision phase (see Fig. 1A), time is required for the rheumatologist to interpret the analysis of the tissue sample and discuss the implications for treatment with the patient (via telephone).

### Resource use and unit costs

The types and quantities of resources and corresponding unit costs for staff time and consumables are reported in Tables 1 and 2, respectively. The duration of time for healthcare staff to complete the tasks within the pre-biopsy and treatment decision post-biopsy phases were estimated by expert input from rheumatologists (F.R., C.P. and A.B.) experienced with the procedure and a patient (Z.I.) with experience in biopsy-driven precision medicine trials. Expert input was obtained during one online videocall with all individuals to reach a consensus on the expected durations for an average patient alongside a plausible upper and lower range (Table 1). Consumables to complete the US-guided synovial tissue biopsy were identified from the non-research procedures detailed in the SOP for the R4RA trial (Table 2). The duration of the biopsy procedure was estimated from the mean observed time to complete tissue collection using individual participant data from the R4RA trial participants who received a needle biopsy (*n* = 83 participants). This mean observed duration was 49.14 min, with an observed range of 15–150 min.

**Table 1.** Resource use and unit cost: staff time.

Action taken	Staff member	Duration (min)		Cost (£)		Source	
		Expected	Range	Cost/min	Average cost	Duration	Unit cost
Pre-biopsy							
Inform patient about procedure and obtain consent	C	5	4–15	£2.38	£11.92	Expert estimation	[21]
Identify joint for biopsy	C	3	2–6	£2.38	£7.15	Expert estimation	[21]
Biopsy procedure							
Complete a biopsy checklist	C	1	0.8–1.2	£2.38	£2.38	Expert estimation	[21]
Trolley set-up	N	5	4–6	£1.15	£5.75	Expert estimation	[21]
Operator antiseptic	N	5	4–6	£1.15	£5.75	Expert estimation	[21]
Orientation of the patient	N	1	0.8–5	£1.15	£1.15	Expert estimation	[21]
Skin sterilization	N	4	3–5	£1.15	£4.60	Expert estimation	[21]
Place US probe within the sterile sheath	N	1	0.8–2	£1.15	£1.15	Expert estimation	[21]
Fluid aspiration	C	2	1.6–3	£2.38	£4.77	Expert estimation	[21]
Partial anaesthesia	C	1	0.8–1.2	£2.38	£2.38	Expert estimation	[21]
Synovial tissue collection	C and N	49.14	15–150	£3.53	£173.63	R4RA trial [12]	[21]
Post-procedure care: dress wound	N	1	0.8–1.2	£1.15	£1.15	Expert estimation	[21]
Post-procedure care: functional assessment	N	2	1–5	£1.15	£2.30	Expert estimation	[21]
Post-biopsy							
Interpretation of biopsy sample results	C	5	3–10	£2.38	£11.92	Expert estimation	[21]
Discussion of the results with patient	C	10	5–15	£2.38	£23.83	Expert estimation	[21]
Write letter to confirm prescribing decision	C	5	3–10	£2.38	£11.92	Expert estimation	[21]

C: consultant; N: nurse; R: registrar.

Unit cost for nurses assumed a Band 7 Advanced Nurse.

**Table 2.** Resource use and unit costs: consumables.

Consumable	Quantity	Cost (£)			Source	
		Unit cost	Range	Average cost	Quantity	Unit cost
Quick Core biopsy needles (16G/21G)	1	£29.35	£23.48–£35.22	£29.35	R4RA trial SOP	Purchase price
19G needles to collect tissue	19	£0.30	£0.24–£0.36	£5.70	R4RA trial SOP	Purchase price
Sterile drapes	1	£6.30	£5.04–£7.56	£6.30	R4RA trial SOP	Purchase price
Sterile gown and gloves	1	£8.50	£6.80–£10.20	£8.50	R4RA trial SOP	Purchase price
Sterile US sheath	1	£1.95	£1.56–£2.34	£1.95	R4RA trial SOP	Purchase price
Sterile dressing pack	1	£0.72	£0.58–£0.86	£0.72	R4RA trial SOP	Purchase price
Absorbent pad	1	£0.22	£0.18–£0.26	£0.22	R4RA trial SOP	Purchase price
Face mask and cap	1	£0.18	£0.14–£0.22	£0.18	R4RA trial SOP	Purchase price
Sterile gauze swabs	3	£1.10	£0.88–£1.32	£3.30	R4RA trial SOP	Purchase price
Non-adhesive dressing	1	£0.07	£0.06–£0.08	£0.07	R4RA trial SOP	Purchase price
Antiseptic solution (100 ml)	1	£2.48	£1.98–£2.98	£2.48	R4RA trial SOP	Purchase price
10 ml syringes	1	£0.04	£0.03–£0.05	£0.04	R4RA trial SOP	Purchase price
20 ml syringes	1	£0.08	£0.06–£0.10	£0.08	R4RA trial SOP	Purchase price
1% lignocaine (20 ml)	1	£1.38	£1.10–£1.66	£1.38	R4RA trial SOP	Purchase price
RNAlater solution (5 ml)	1	£11.58	£9.27–£13.90	£11.58	R4RA trial SOP	[25]
US biopsy machine	2	£6.32	£5.06–£7.58	£12.64	R4RA trial SOP	[22–24]

Purchase price obtained from the Centre for Experimental Medicine and Rheumatology, Queen Mary University of London.

The unit costs of staff time were obtained from the national pay scales for healthcare staff published in the Unit Costs for Health and Social Care [21]. The unit cost per patient for a US scan (£6.32) was estimated by apportioning the reported capital cost (£65 000) over an expected equipment lifetime of 7 years, with annual maintenance requiring 6% of the total capital cost per year, and an operating capacity of 1 scan/hour over a 40-hour working week [22–24]. The unit cost of consumables for the biopsy procedure were obtained from the purchase price reported by the Centre for Experimental Medicine and Rheumatology, Queen Mary University of London (consumables obtained via NHS Procurement) and public-facing list prices [25]. Unit cost estimates for consumables are presented as their purchase price with 20% variation to define a plausible range (Table 2).

### Data analysis

The primary outcome was reported as the average (mean) cost per patient of one US-guided synovial tissue biopsy. This average cost was calculated by multiplying the quantity of resources consumed by the corresponding unit costs and summing across all levels of activity. The base case estimate used all resource and unit costs at their average values. One-way sensitivity analysis was performed by adjusting each unit cost and resource estimate independently to its upper and lower value [26]. The results from the one-way sensitivity analysis were presented as a tornado diagram to illustrate which components were the key drivers of cost per patient [27]. A best-case and worst-case analysis re-estimated the average cost per patient with all cost and resource estimates set to their lowest and highest values, respectively. Two scenario analyses were performed to investigate how the average cost per patient will change under alternative models of service delivery. Scenario A assumed that a registrar would perform the biopsy procedure instead of a consultant medical doctor to reflect clinical scenarios when less senior staff members acquire synovial tissue samples. The unit cost of a registrar was £1.22/min of activity [21]. Scenario B assumed that an additional outpatient appointment was required pre-biopsy to facilitate joint identification, which reflected a clinical scenario when US capacity is not available within a routine outpatient setting. The unit cost of an outpatient US appointment was obtained from the National Cost Collection data [28].

### Ethics

All participants in the R4RA trial provided written informed consent. The study was done in compliance with the Declaration of Helsinki, International Conference on Harmonization Guidelines for Good Clinical Practice and local country regulations. The protocol was approved by the institutional review board of each study centre or relevant independent ethics committee (UK Medical Research and Ethics Committee, reference no. 12/WA/0307).

## Results

The results are reported in Table 3. In the base case analysis, the total cost of US-guided synovial tissue biopsy was £356.24/patient. The biopsy phase contributed the most to this cost (£283.18/patient; 79% of the total cost), followed by the post-biopsy treatment decision phase (£47.67/patient; 13% of the total cost) and the pre-biopsy phase (£25.39/patient; 7% of the total cost).

**Table 3.** Cost of synovial tissue biopsy: base case and scenario analyses.

Scenario	Total cost (£)
Base case analysis	356.24
Input parameter sensitivity analyses	
Best-case sensitivity analysis	185.30
Worst-case sensitivity analysis	812.46
Structural scenario analyses	
A: Registrar doctor performs the biopsy procedure	294.24
B: Additional outpatient US appointment required to select joint	438.98

### Sensitivity analysis

**Figure 2.**
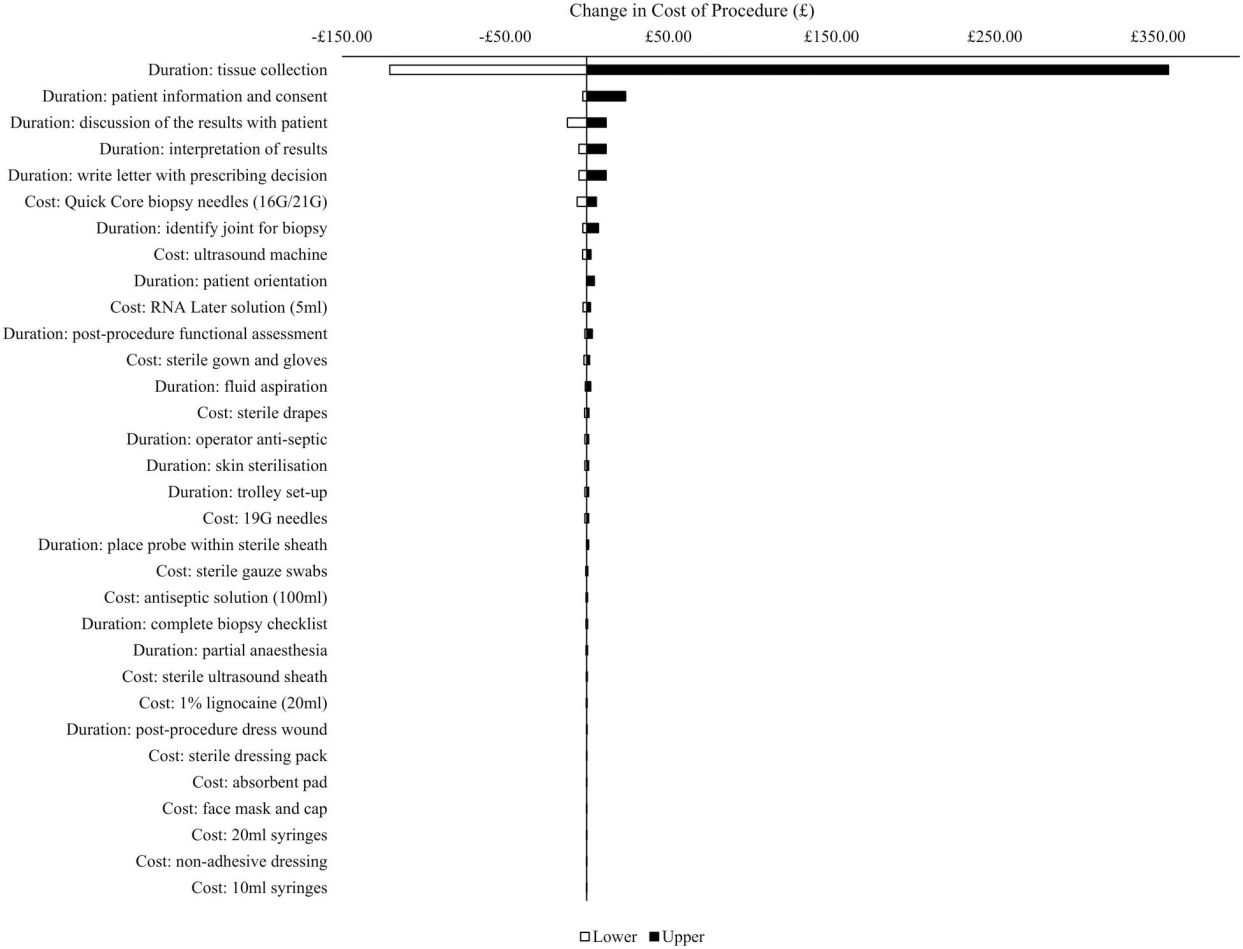
One-way sensitivity analysis of input parameter values on total cost. Light and dark bars refer to the change in the cost of synovial tissue biopsy when input parameter values are adjusted to their lower and upper plausible values, respectively

Fig. 2 reports a tornado diagram to summarize the results of the one-way sensitivity analysis. The greatest driver of cost per patient was the duration of time taken to undertake synovial tissue collection. By varying this duration estimate to its upper and lower plausible values, the total cost per patient ranged between £235.61 and £712.61. Four of the remaining key drivers of total cost were parameters that measured the duration of healthcare staff time. By varying all resource and cost estimates to their lower and upper values simultaneously, the respective best-case and worst-case estimates of total cost per patient were £185.30 and £812.46.

### Scenario analysis

The results of the two scenario analyses are reported in Table 3. For scenario A, by assuming that a registrar will complete the tissue sample collection procedure, the total cost per patient (£294.24) was £62 lower than the base case estimate. For scenario B, by assuming that an additional outpatient visit is required to perform a US scan for identifying whether a joint is suitable for biopsy, the total cost per patient (£438.98) was £82.74 higher than the base case estimate.

## Discussion

Information gained from synovial tissue biopsies offers a promising way to improve management decisions for people with RA [3]. This study found that one synovial tissue biopsy will cost £356.24/patient. Emerging evidence indicates that information obtained from synovial tissue samples may inform long-term prognosis and response to downstream treatments [9]. Care providers will be able to use evidence of heterogeneity between people with RA to improve care as new effective management strategies, informed by the analysis of synovial tissue samples, start to enter routine care. To support the translation of biopsy-based management strategies into standard care, health economic data are required, in addition to effectiveness data, to inform resource allocation decisions for RA [19]. The findings presented by this study deliver the necessary evidence to help care providers understand the cost and resource impact of adopting synovial tissue biopsies within their own care settings.

Understanding the required resources and potential cost of performing a synovial tissue biopsy is an essential first step towards establishing the cost-effectiveness of this procedure within the standard of care. The cost-effectiveness of this procedure will be determined by how the information obtained from the acquired tissue sample will inform a subsequent management decision [29]. The bottom-up micro-costing approach reported by this study demonstrated the importance of going beyond the direct cost of consumables incurred to examine how to embed biopsy procedures within established pathways of care, the staff time for delivering these additional care processes and the potential turnaround times between sample acquisition, analysis and feedback. The sensitivity analyses indicated potential targets to optimize the cost of performing a synovial tissue biopsy. For example, the duration of sample acquisition was a key driver of total cost that may decrease over time through greater provider experience or substituting towards interventional radiologists skilled with performing biopsy-based procedures [30]. The scenario analysis indicated that reducing the need for an additional patient visit to perform a US examination for joint identification will also reduce the total cost of the procedure. However, this will require spare capacity in US devices during routine outpatient consultations, which may vary between provider centres.

While alternative estimates for the cost of a synovial tissue biopsy were not available in the literature, the cost of similar procedures may be helpful instead to contextualize the findings from this study. For example, according to the National Schedule of NHS Costs, the estimated cost of a US-guided core needle biopsy of a breast lesion was £490 (currency code: YJ13Z), which is similar to the base case cost estimated by the present study [28]. Alternatively, the cost of an endoscopic US examination with biopsy was £1217 (currency code: GB12Z), which is greater than the base case cost estimate [28]. The complexity and duration of a US-guided synovial tissue biopsy falls between a core needle biopsy and an endoscopy. The costs of these similar procedures suggests that the results from the present study have face validity within a rheumatology setting.

The outputs from this analysis can be used in several ways. First, the estimated cost of the biopsy procedure can be used by future health economic evaluations aiming to estimate the costs and health benefits of different candidate intervention procedures that use information obtained from synovial tissue samples. Second, care providers can scale up the upfront cost of the biopsy procedure by their anticipated patient volume within a financial year to inform budget impact assessments or business cases for adoption within rheumatology services [31]. Third, healthcare systems can use the estimated cost from this study to inform unit of activity reimbursement tariffs to support the wider provision of synovial tissue biopsies [32]. Finally, the outputs can be used to inform the potential cost of synovial tissue biopsies in different countries. To achieve this, decision makers will first need to adapt the service pathway conceptual model, if required, to reflect the provision of care within their own country and assign unit costs relevant for their healthcare system.

One limitation of this study was that the estimated time to perform the biopsy procedure was taken from direct measurement within a clinical trial setting. This may have underestimated the time required to perform biopsies within routine care because the providers who contributed to the trial itself may be specialized in the procedure. Alternatively, these duration data from a trial setting may overestimate the time expected within routine care because the synovial tissue biopsies were research activities performed without the time constraints of a standard clinical setting. To handle this uncertainty, the results reported the estimated total cost across the range of durations observed within the supporting trial data, so decision makers can explore the corresponding impact of longer and shorter durations within a routine care setting. A second limitation was that expert opinion was required to estimate the time for clinical activities before and after the biopsy procedure. However, to mitigate the impact on the estimated total cost, input was sought from multiple rheumatologists and a patient representative. A plausible range of input parameter values was included to explore this parameter uncertainty within the sensitivity analysis. A third limitation was that the unit costs for healthcare resources were measured from the perspective of the healthcare system in England, which may not generalize to other countries with different cost structures. However, the quantities of resources consumed are likely to generalize internationally because the SOP for the US-guided synovial tissue biopsy within the R4RA trial was used in different countries, including Belgium, Italy, Portugal and Spain.

Future research could undertake qualitative research with rheumatology care providers and people with RA to explore potential barriers to delivering synovial tissue biopsy within routine care. In addition, this study can be replicated in different countries to estimate the cost of synovial tissue biopsy and the key drivers of total cost within different healthcare jurisdictions. Finally, future research should use the results from this study within cost-effectiveness analyses of intervention strategies that require a synovial tissue biopsy to estimate the value of these procedures, in terms of cost and health outcomes, compared with current clinical practice.

## Conclusion

There is growing international interest in using synovial tissue biopsies to inform management decisions for people with RA. At a practical level, care providers and decision makers will need to have a reliable understanding of the resource requirements and cost of this procedure before it can be adopted within the standard of care. The findings from this study provide this essential cost evidence to help translate synovial tissue biopsies into clinical practice. Given that the steps required to perform a synovial tissue biopsy are generalizable internationally, the necessary resource requirements estimated by this study will also generalize beyond a UK setting. As effective intervention strategies supported by analysing synovial tissue samples start to emerge, these findings will ultimately help care providers deliver these interventions within their resource-constrained environment and people with RA to benefit from their wider implementation.

## Data Availability

The non-individual participant data underlying this article are available in the article. The anonymized individual participant data underlying this article are stored in a non-publicly available repository. All data requests should be submitted to the corresponding author of the original R4RA trial manuscript for consideration. Access to anonymized data may be granted following review.
